# Supplementary material for: Controlled hydrogenation into defective interlayer bismuth oxychloride via vacancy engineering
Source: Commun Chem. 2020 Jun 5;3:73. doi: 10.1038/s42004-020-0319-9 (PMC9814713; doi:10.1038/s42004-020-0319-9)
Supplement: Supplementary file 1 — Supplementary Information [file 42004_2020_319_MOESM1_ESM.pdf]

Controlled Hydrogenation into Defective Interlayer Bismuth oxychloride via Vacancy Engineering

Dandan Cui[1,2], Kang Xu[1], Xingan Dong[3], Dongdong Lv[1], Fan Dong[3], Weichang Hao[1], Yi Du[1,4], and Jun Chen [2]

[1]School of Physics and BUAA-UOW Joint Research Centre, Beihang University, Beijing 100191, China

[2]ARC Centre of Excellence for Electromaterials Science (ACES), Intelligent Polymer Research Institute (IPRI) and, Australian Institute for Innovative Materials (AIIM), University of Wollongong, Wollongong, NSW 2500, Australia

[3]Research Center for Environmental Science & Technology, Institute of Fundamental and Frontier Sciences, University of Electronic Science and Technology of China, Chengdu 611731, China

[4]Institute for Superconducting and Electronic Materials (ISEM), Australian Institute for Innovative Materials (AIIM), University of Wollongong, Wollongong, NSW 2500, Australia

Supplementary Methods

Chemicals and Gases. Bismuth (III) nitrate pentahydrate ($Bi(NO_3)_3 \bullet 5H_2O$), sodium chloride (NaCl), Sodium sulphate (Na_2SO_4), ethylene glycol (EG) and hydrochloric acid (HCl) (36%) and ethanol were all purchased from Sigma Aldrich. Deionized water was taken from a Millipore Autopure system. All chemicals were used without further purification. Mixed hydrogen and argon was also used (H_2 :Ar=1:9), with both gases 99.999% purity.

Synthesis of BiOCl with oxygen vacancy. In a typical synthesis, 1 mmol bismuth (III) nitrate pentahydrate and 1 mmol sodium chloride were dissolved in 15ml ethylene glycol solution with vigorous stirring for 30 min. Then 0.5 mL hydrogen chloride was added into the above solution. The mixed solution was transferred to 20 ml Teflon-lined stainless-steel autoclave, which was kept in an oven at 150 ºC for 15 h. After the solvothermal reaction, the same volume of de-ionized water was added into the resulting solution then under vigorous magnetic stirring for 0.5 h at room temperature. The obtained product was collected by centrifugation and washed several times with deionized water and ethanol to remove residual ions. The resultant composite was dried at 80°C for 12 h for further characterization, with the final product denoted as “BiOCl oxygen vacancy” (or BiOCl OV).

Synthesis of hydrogen modified BiOCl oxygen vacancy. The hydrogen-modified BiOCl OV (H-BiOCl OV) sample was prepared by annealing BiOCl OV, putting 1g BiOCl OV in a porcelain boat, placed in a tubular furnace, sintered from room temperature to 200 ºC under hydrogen and argon gas mixture (at a heating rate of 10 ºC min^{-1}) and then dwelling for 3h at 200 ºC before cooling.

Supplementary Figures

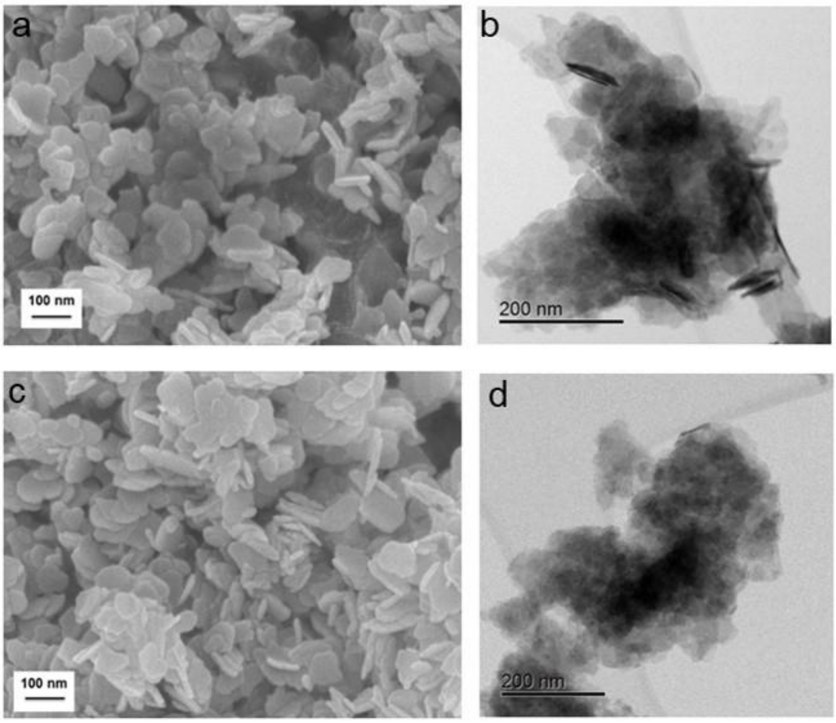

Supplementary Figure 1 SEM images of (a) H-BiOCl OV and (d) BiOCl OV; TEM images of (b) H-BiOCl OV and (d) BiOCl OV.

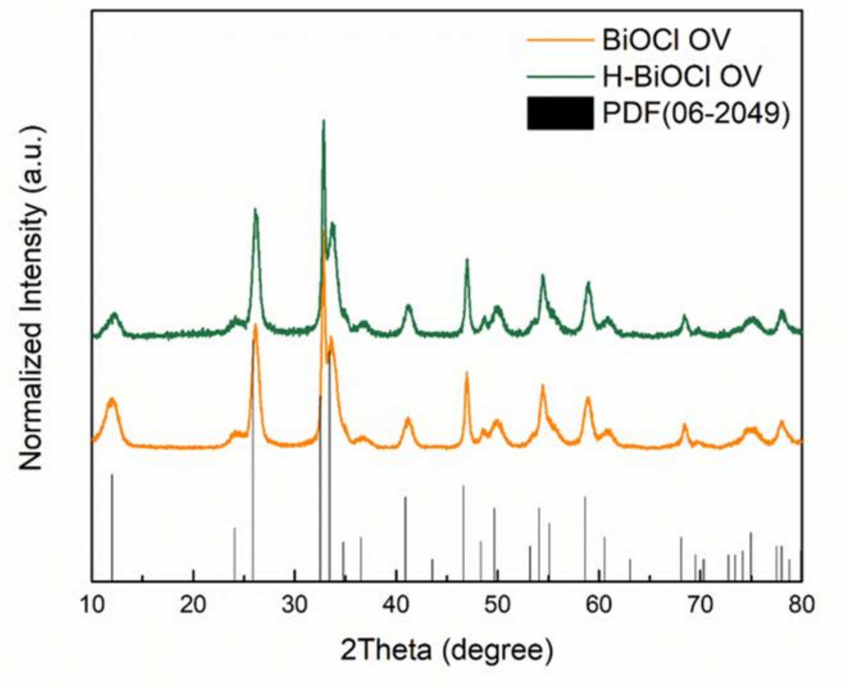

Supplementary Figure 2 XRD pattern of BiOCl OV and H-BiOCl OV.

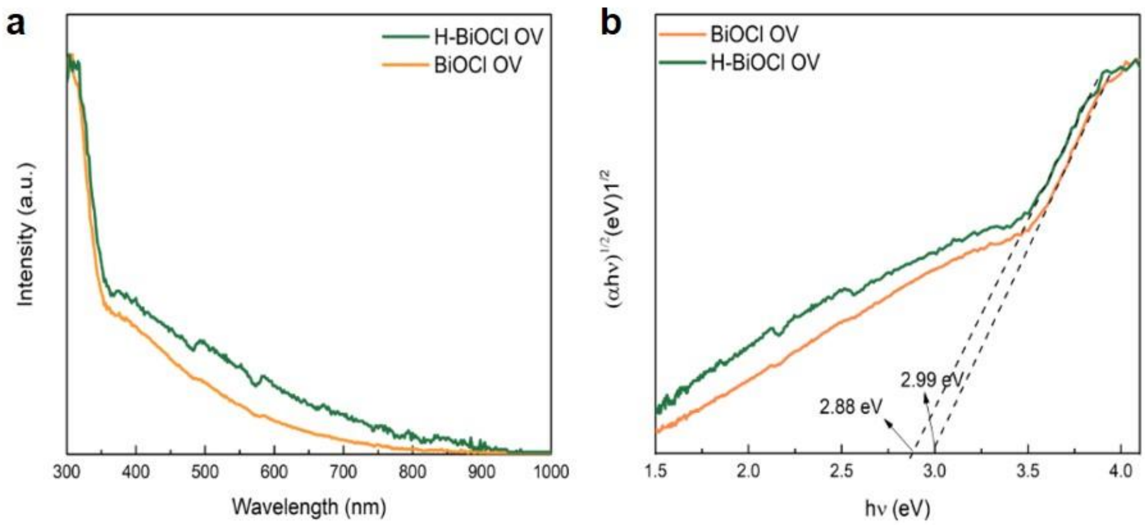

Supplementary Figure 3 (a) UV–vis diffuse reflectance spectra of BiOCl OV and H-BiOCl OV; and (b) Optical band gaps of BiOCl OV and H-BiOCl OV determined by UV-vis diffuse reflectance spectroscopy.

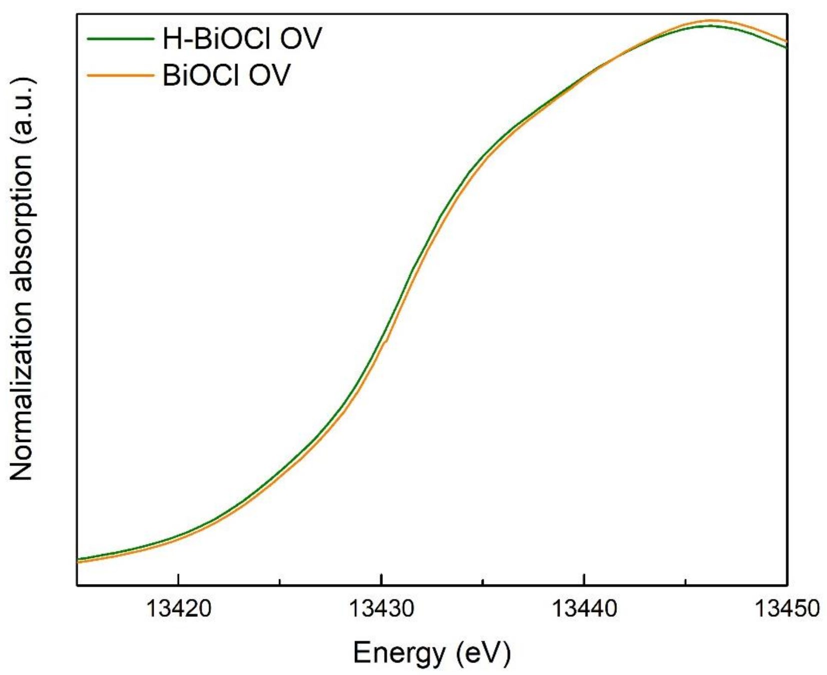

Supplementary Figure 4 XANES spectra at the Bi L-edge.

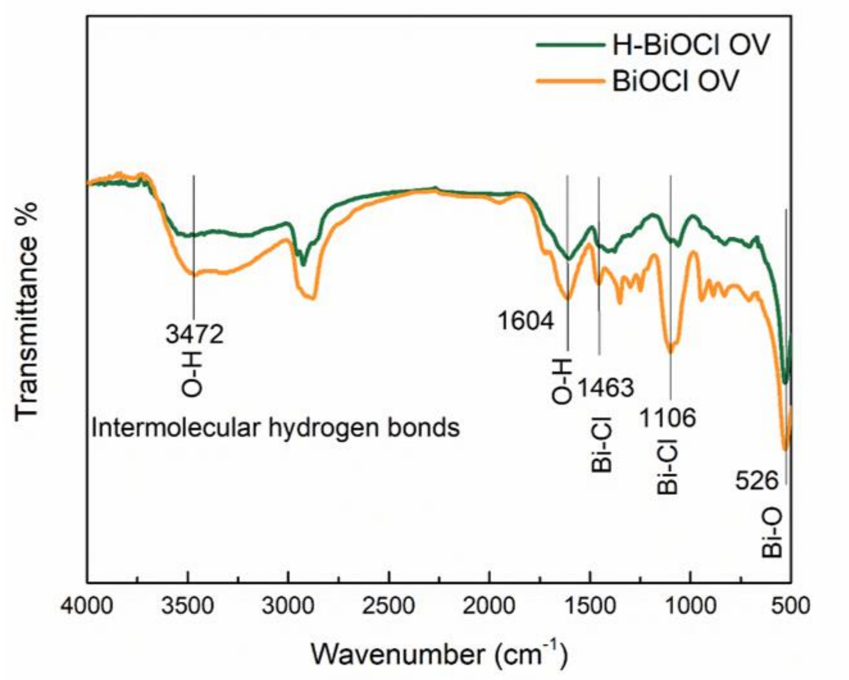

Supplementary Figure 5 DRIFTS spectra of BiOCl OV and H-BiOCl OV.

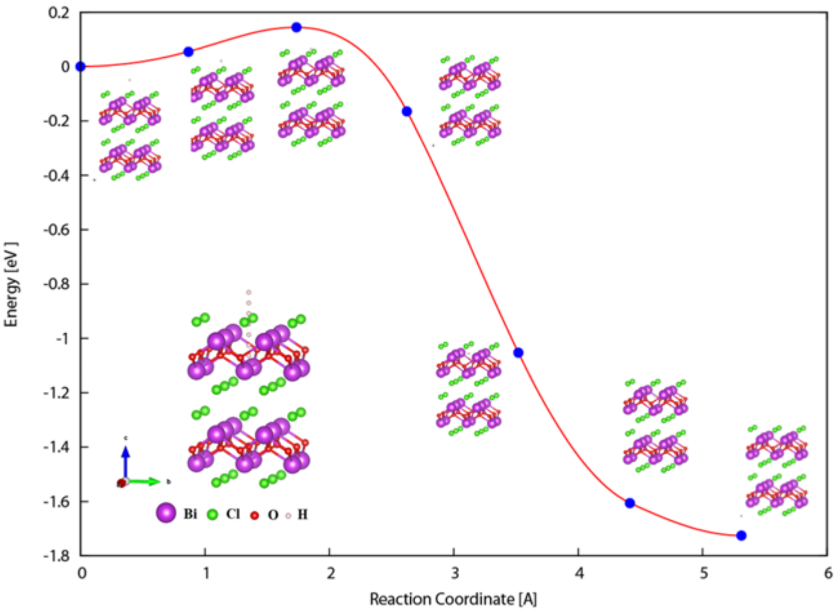

Supplementary Figure 6 Reaction energy paths for H atom dissociation, upper panel, and H atom displacement along of the BiOCl OV (001) direction, lower panel. Insets show the geometries of the systems at the relevant points in the paths

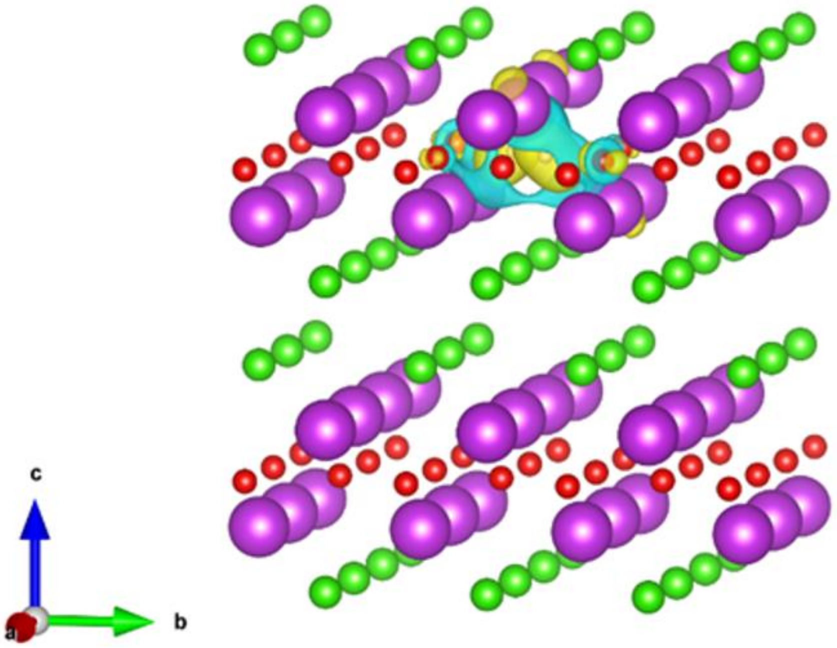

Supplementary Figure 7 The calculated difference charge density contour of H-BiOCl OV.

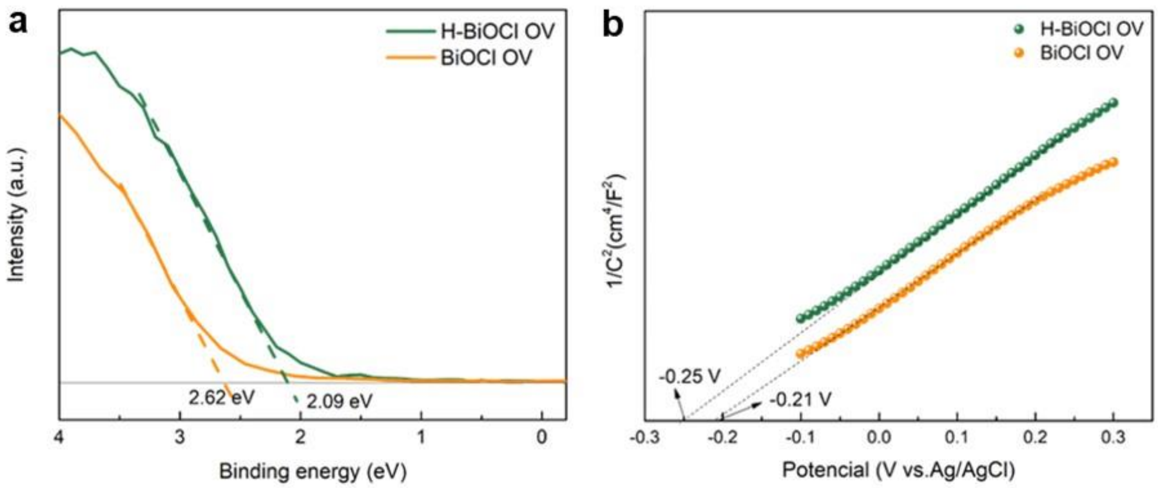

Supplementary Figure 8 (a) XPS valance band spectra and (b) Mott-Schottky plots of BiOCl OV and H- BiOCl OV

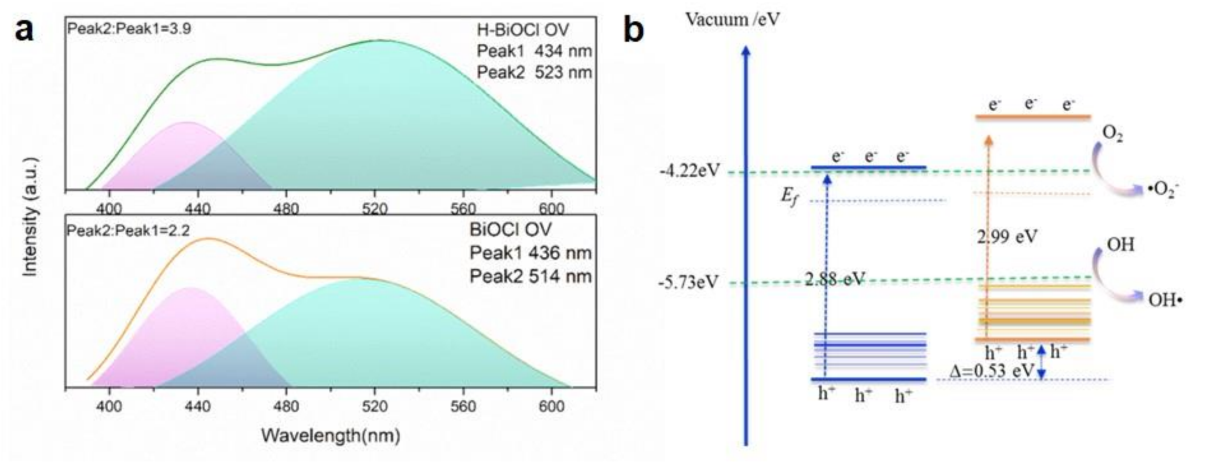

Supplementary Figure 9 (a) Photoluminescence spectra of samples and (b) Schematic illustration of the energy band alignment of BiOCl OV and H- BiOCl OV.

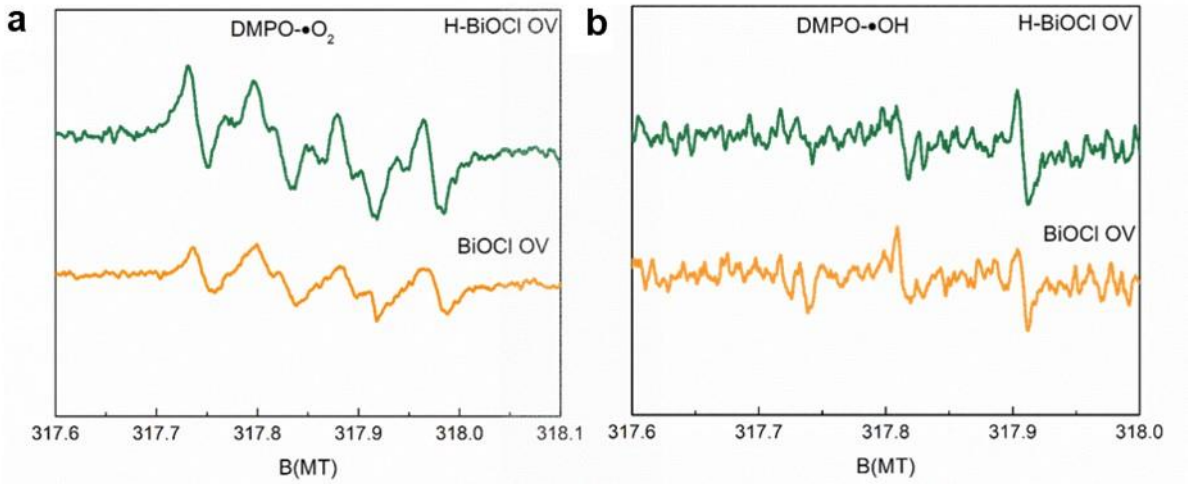

Supplementary Figure 10 DMPO spin-trapping spectra of H-BiOCl OV and BiOCI OV (a) in methanol dispersion for DMPO-•O_2 (b) in aqueous dispersion for DMPO-•OH

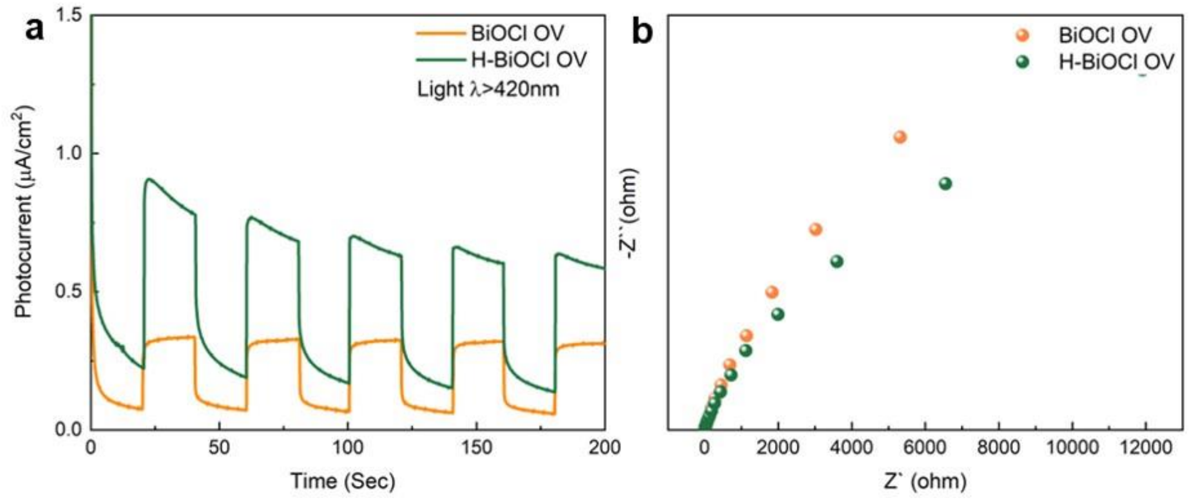

Supplementary Figure 11 (a) Transient photocurrent responses of of BiOCl OV and H-BiOCl OV. (b) Quist plots for the H-BiOCl OV H-BiOCl OV samples under visible light irradiation (λ ≥ 420 nm).

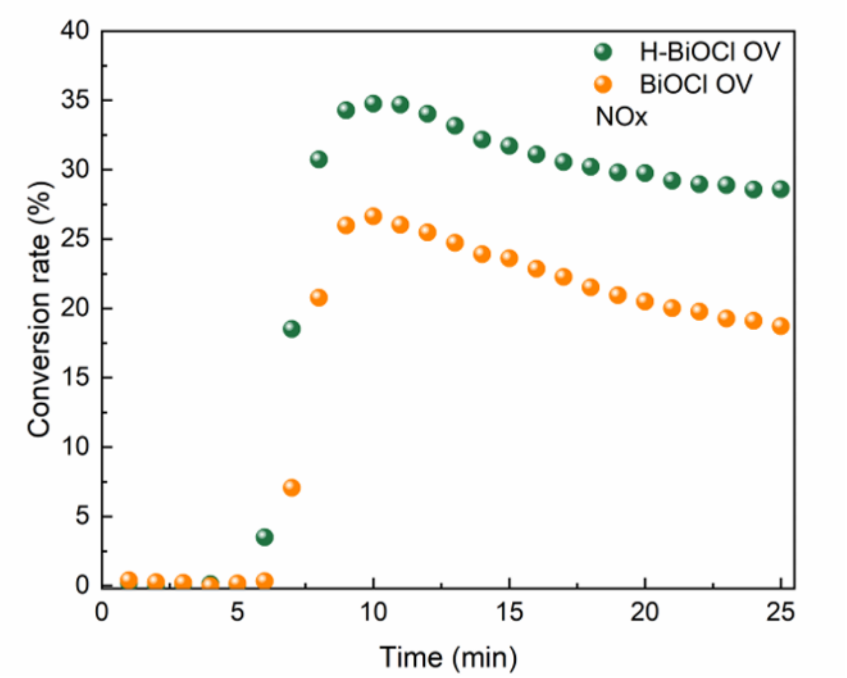

Supplementary Figure 12 NO conversion rate for NOx production in the presence of BiOCl OV and H-BiOCl OV.

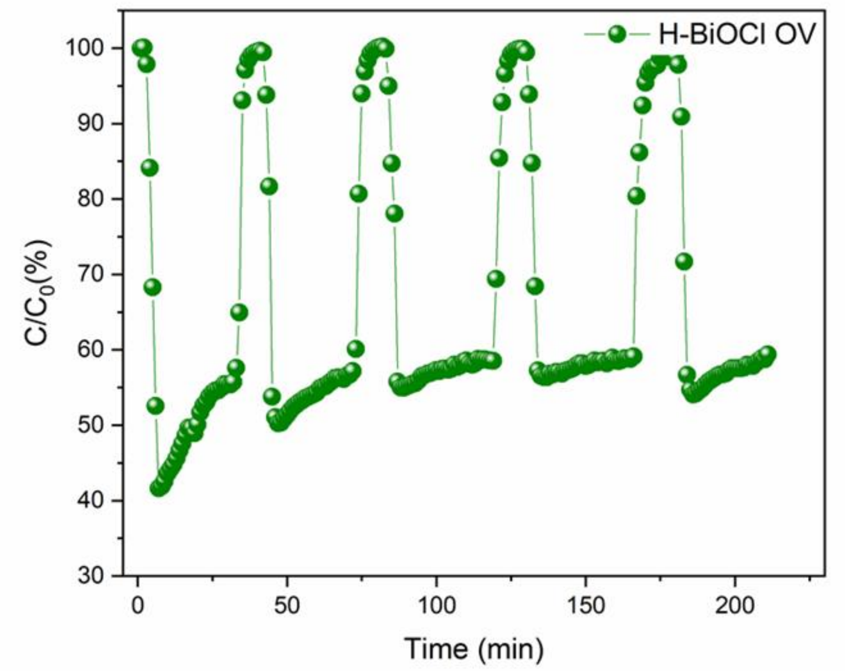

Supplementary Figure 13 Cycling measurements of photocatalytic NO oxidation with H-BiOCl OV.

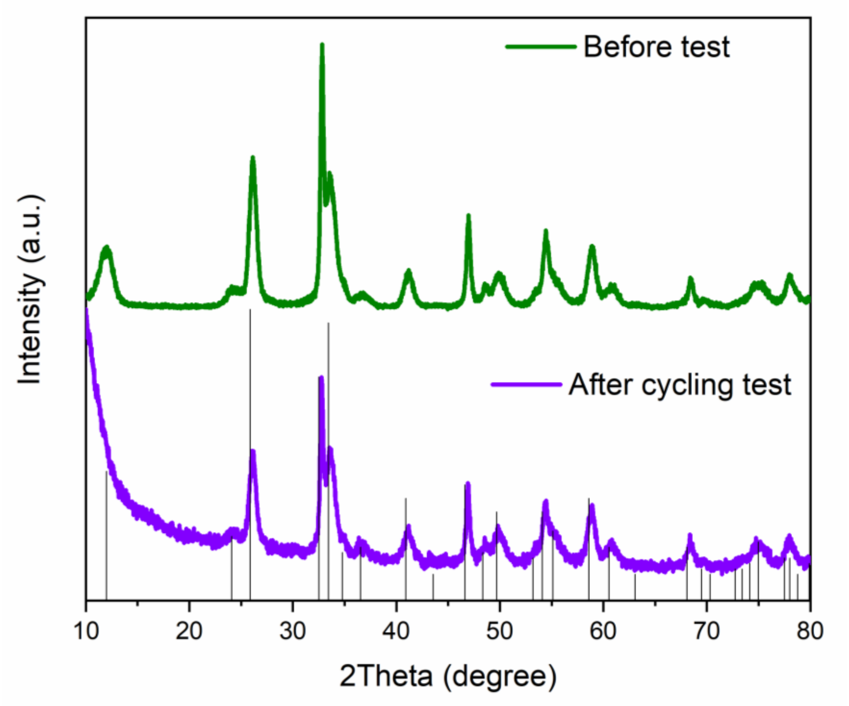

Supplementary Figure 14 XRD patterns of H-BiOCl OV before and after the cycling test of NO oxidation under visible light.

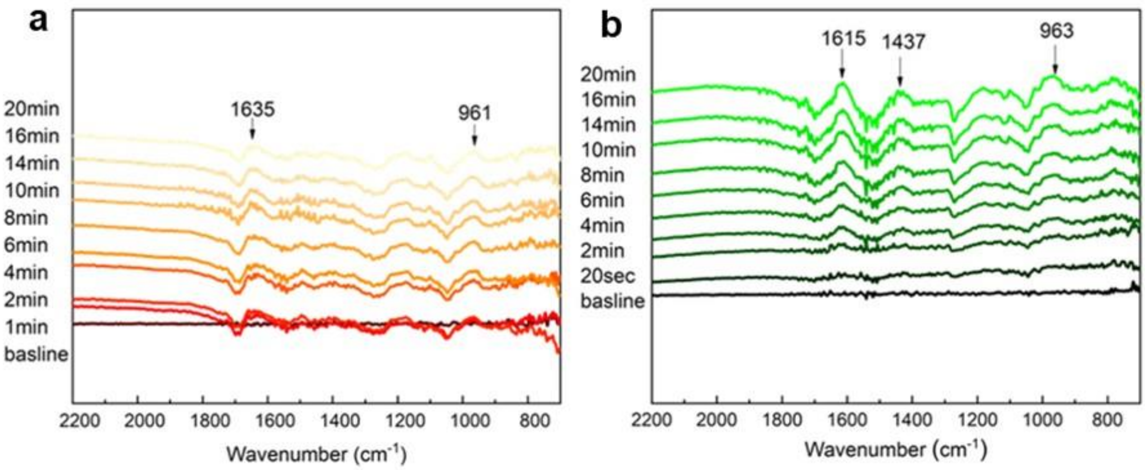

Supplementary Figure 15 (a), (b) *in-situ* FT-IR spectra photocatalytic adsorption process of NO on the surface of BiOCI OV and H-BiOCI OV under dark.

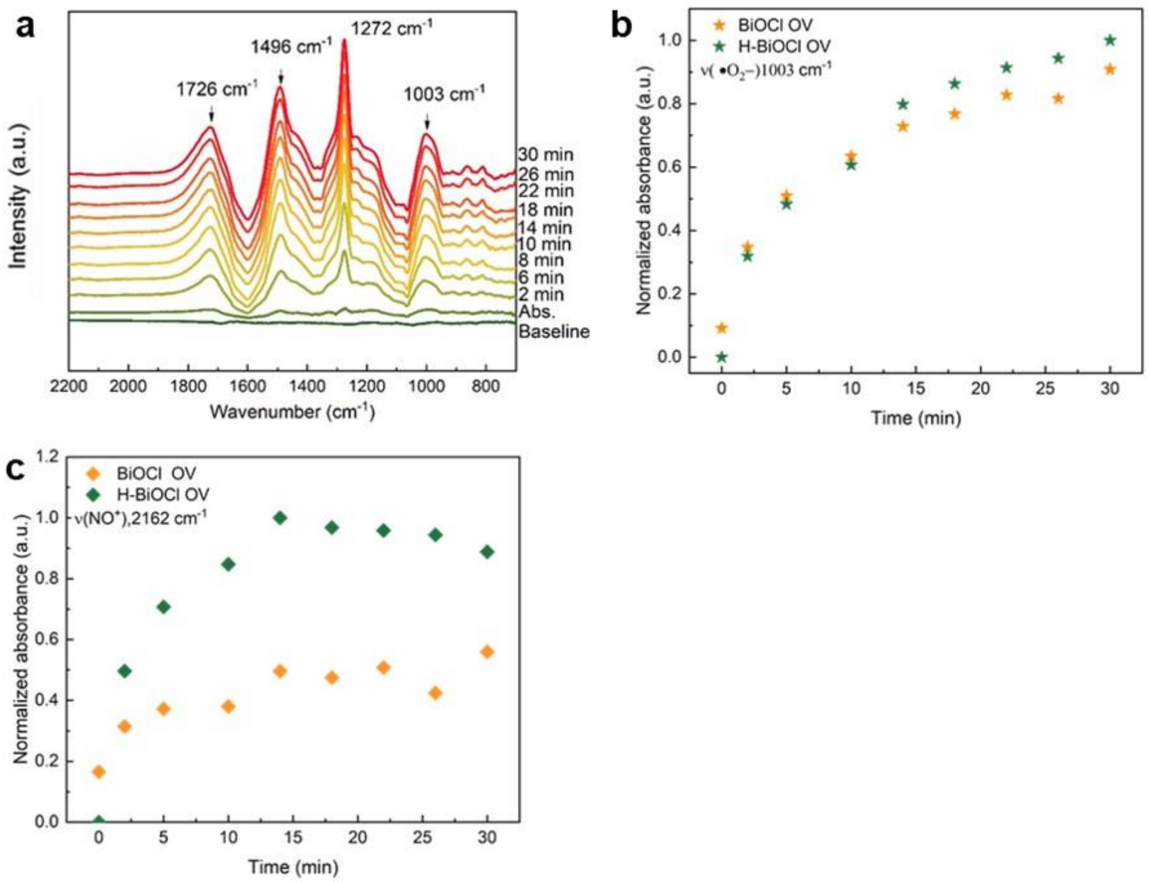

Supplementary Figure 16 *In-situ* FT-IR spectra of the degradation of 50% NO + 50% O_2 of BiOCl OV at the same time; (b); The corresponding normalized absorbance curves of H-BiOCl OV and BiOCl OV at ν(•O_2-) 1003 cm^{-1} (c) and ν(NO^+) 2162 cm^{-1} (d).

Supplementary Discussion

Through the analysis data, the possible reaction mechanism of NO photocatalytic oxidation by H-BiOCl OV can be proposed as follows:

$$hv\ (visible\ light) \longrightarrow h^+ + e^- \quad (1)$$

$$e^- + O_2 \longrightarrow \cdot O_2^- \qquad h^+ + OH^- \longrightarrow \cdot OH \quad (2)$$

Reaction pathway 1:

$$NO + O_2 \longrightarrow NO_2 \quad (3)$$

$$NO_2 + e^- \longrightarrow NO_2^- \quad (4)$$

$$2NO_2^- + \cdot O_2^- + h^+ \longrightarrow 2NO_3^- \quad (5)$$

Reaction pathway 2:

$$NO + \cdot OH \longrightarrow OH^- + NO^+ \quad (6)$$

$$NO^+ + \cdot O_2^- + e^- \longrightarrow NO_3^- \quad (7)$$
